# Endovascular embolization for basal ganglia and thalamic arteriovenous malformations

**DOI:** 10.3389/fneur.2023.1244782

**Published:** 2023-11-02

**Authors:** Jing Lan, Yi-hui Ma, Yu Feng, Ting-Bao Zhang, Wen-yuan Zhao, Jin-cao Chen

**Affiliations:** Department of Neurosurgery, Zhongnan Hospital of Wuhan University, Wuhan, China

**Keywords:** basal ganglia, thalamus, arteriovenous malformation, transarterial approach, transvenous approach

## Abstract

**Background:**

Basal ganglia and thalamic arteriovenous malformations (AVMs) represent a special subset of malformations. Due to the involvement of vital brain structures and the specifically fine and delicate angioarchitecture of these lesions, it presents unique therapeutic challenges and technical difficulties that require thorough treatment planning, individualized treatment strategies, and advanced techniques for good clinical outcome.

**Method:**

In this study, we presented a series of ruptured basal ganglia and thalamic AVMs embolized via a transarterial, transvenous or combined approach. Herein, we summarized our treatment experience and clinical outcomes to further evaluate the effectiveness and safety of endovascular embolization for these AVMs as well as the indications, therapy strategies, and techniques of embolization procedures.

**Results:**

Twelve patients with basal ganglia and thalamus AVMs were included in the study. Their average age was 23.83 ± 16.51 years (range, 4–57 years) with a female predominance of 67% at presentation. The AVMs were located in the thalamus in 3 (25%) patients, in the basal ganglia in 3 (25%) patients, and in both sites of the brain in 6 (50%) patients. There were 5 AVMs located on the left side and 7 on the right. The mean nidus diameter was 3.32 ± 1.43 cm (range 1.3–6.1 cm). According to the Spetzler-Martin grading classification, 4 (33.3%) brain AVMs were Grade III, 7 (58.3%) were Grade IV, and 1 (8.3%) was Grade V. All of them presented with bleeding at admission: four of these patients presented with an intracerebral hemorrhage (ICH), 8 ICH in combination with intraventricular hemorrhage (IVH), and no patient with subarachnoid hemorrhage (SAH). Among these patients treated with endovascular embolization, 7 patients were treated by the transarterial approach, 4 patients transvenous approach, and 1 patient underwent the combined approach. A single embolization procedure was performed in 6 patients (50%) and the other 6 cases (50%) were treated in a staged manner with up to three procedures. Procedure-related complications occurred only in two patient (16.7%). Complete AVM obliteration was obtained in 7 patients (58.3%), and partial obliteration was in 4 patients (33.3%). Overall, good or excellent outcomes were obtained in 7 patients (58.3%), and poor functional outcome was observed in 5 patients (41.7%) at the last follow-up. All survived patients achieved anatomic stabilization and there was no postoperative bleeding or recurrence in the follow-up.

**Conclusion:**

The management of the basal ganglia and thalamic AVMs is a great challenge, which needs multimodal individualized treatment to improve the chances of radiographic cure and good outcomes. Endovascular therapy is safe and effective in the treatment of cerebral AVMs particularly for deep-seated AVMs such as the basal ganglia and thalamus. Our results demonstrate a high rate of anatomic obliteration with an acceptable rate of complications in the endovascular treatment of these vasculopathies via a transarterial approach or a transvenous approach.

## Introduction

AVM incidence is 0.69–1.32 per 100,000 person-years (1) in that basal ganglia and thalamic AVMs constitute 4 to 11% of all AVMs (2). Deep-seated AVMs often involve important structures such as the basal ganglia and thalamus have a higher annual bleeding rate, morbidity, and mortality risk than superficial AVMs (3, 4) with an annual hemorrhage rate of approximately 9.8%, nearly three- to four-fold greater than that observed for patients with AVMs in all other locations (3). Once an AVM in the basal ganglia or thalamus ruptures, it can lead to catastrophic consequences. Moreover, the morbidity of hemorrhages was reported to be higher for these lesions, 30 to 85.5% of the surviving patients were left with permanent neurological deficits after hemorrhage (5–7) and mortality rates may even reach 42 to 71% (5, 6, 8, 9). Given the devastating effects of hemorrhage from these lesions, the patient suffered AVM in basal ganglia and thalamic should be actively treated to avoid further bleeding.

The treatment goal of brain AVM is the elimination of the nidus and the preservation of the patient’s functional status. However, for basal ganglia and thalamic AVMs, the risk of intervention remains high, and it will add to the initial hemorrhage-related morbidity, with a rate of around 20% of bad outcomes with mRS > 2 in the surviving patients (5). There is little agreement regarding the optimal treatment modality for patients. The treatment strategies and choices are difficult and variable, due to their deep location and unknown etiology as well as the complex angioarchitecture of these lesions. Under the current circumstances, we use endovascular therapy to manage this type of refractory AVMs. The efficacy and safety of transarterial and transvenous embolization of this kind of lesions are still unascertained and the indications for basal ganglia and thalamic AVMs in patients with a history of hemorrhage must be carefully determined, because of the limited number of reports on this subject. In order to further elucidate these issues, the authors retrospectively analyzed the clinical outcomes concerning their series of basal ganglia and thalamic AVMs treated by embolization and evaluated the endovascular curability and feasibility.

## Methods

### Data collection

The data of baseline demographic, clinical, and angiographic informations regarding patients with a cerebral arteriovenous malformation was retrospectively collected and analyzed from 2017 to 2022. Patients with basal ganglia and thalamic AVMs treated by endovascular embolization in the first intention were included in this study. Patients with any other cerebrovascular lesions or incomplete clinical data were excluded. Among them, 12 patients with an AVM involving the basal ganglia and thalamus structures met the above criteria in our institution. Treatment strategies and choices were discussed with a multidisciplinary team including neurosurgeons and neurointerventionists. Written informed consent for our procedure was obtained from individuals or minors’ legal guardians/next of kin.

Demographic data recorded for each patient included age, sex, initial clinical presentation, and functional status. In the case of hemorrhagic presentation, the location of the bleeding was noted (subarachnoid, intraventricular, intracerebral). All patients underwent superselective DSA before the treatment to evaluate the characteristics of vascular lesions. Characteristics of the nidus (size, location, and type of nidal arteriovenous shunts or the morphology of the vascular spaces around the nidus), feeding arteries (number, origin), the extent of the AVM, and venous drainage pathways and pattern were precisely recorded. The volumetric size of the AVM nidus was assessed by the maximal diameters in each of the three orthogonal planes. Comparisons were then made between volumes before and after embolization to estimate the percentage of each AVM that had been occluded. Head CT examination was performed to confirm the presence of hematoma and MRI was used to identify the important information about the relationship of the nidus to the internal capsule, its anatomical location, and the brain edema. The Spetzler-Martin grade was also calculated in every case.

### Outcome evaluation

Clinical outcome was assessed based on post-treatment angiograms and graded as complete or partial obliteration. Complete AVM cure was affirmed by the absence of any early venous drainage at DSA after endovascular treatment. Postoperative deficits were recorded, a hemorrhagic complication was confirmed by CT at the end of the procedure, and postoperative MRI was performed to evaluate ischemic lesions. Follow-up DSA was performed at 6 months after embolization. In cases of residual AVM, any further treatments and DSA checks were dealt with on a case-by-case basis. Functional status was assessed using the mRS, and the pre-treatment mRS was obtained from hospital admission physical examination. Follow-up information was obtained either by telephone interviews with the patient or family members or during a physical examination of those who were able to visit our hospital. We noted the mRS at the time of each visit and angiography. A good outcome was defined as a mRS of 0–2 at the last clinical follow-up and a bad outcome as a mRS of 3–6. Functional improvement was defined as a decrease in the mRS score from the preoperative exam to the final follow-up exam. Deterioration was likewise defined as an increase in the mRS score.

### Transarterial approach

**Figure 1:**
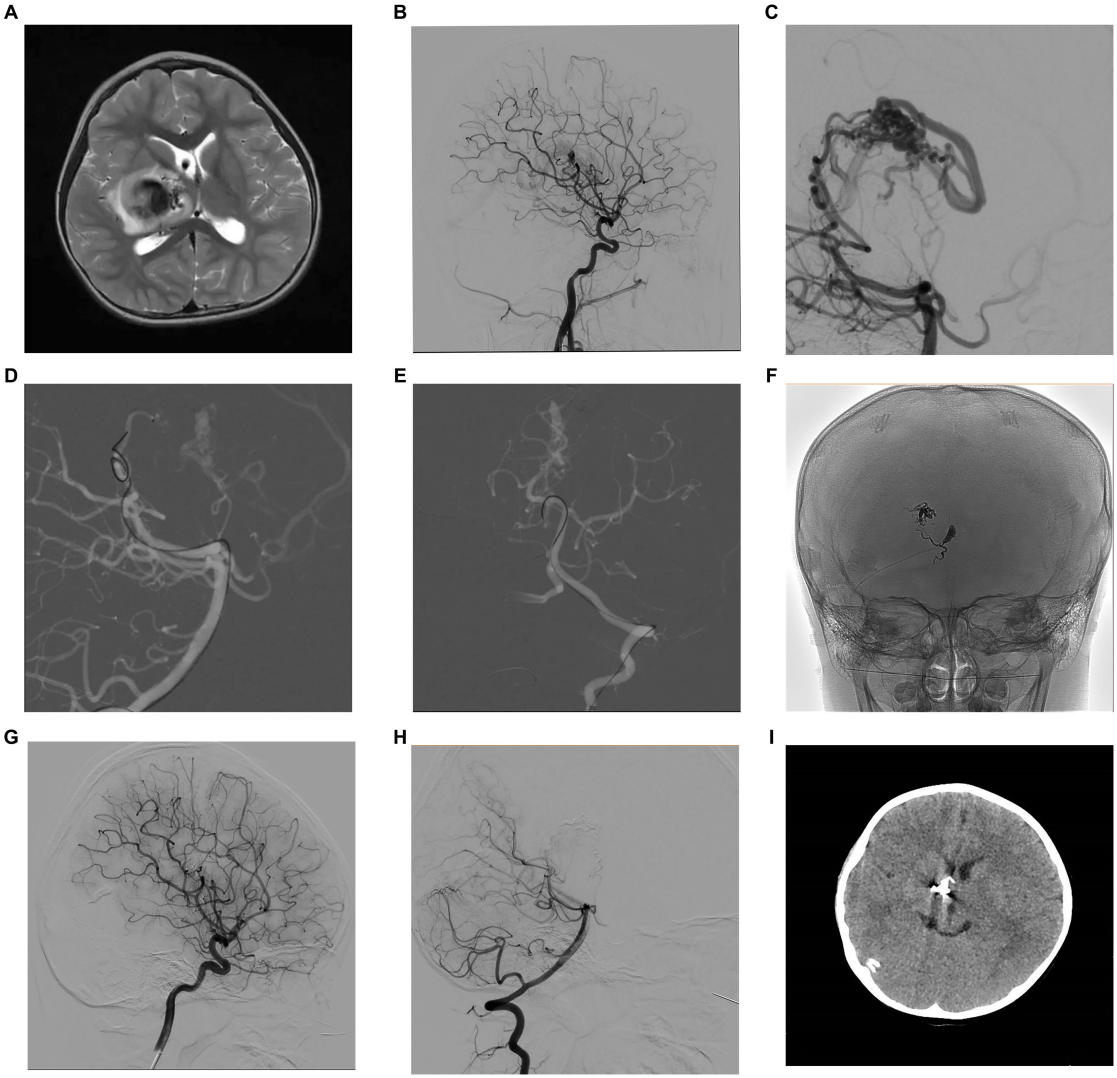
A 4-year-old female patient presented with left limb weakness. Axial T2-weighted MRI **(A)** showed an AVM located in the right basal ganglia and thalamic area. Selective DSA of the right ICA in lateral **(B)** view and vertebral artery in lateral **(C)** projection unfolded an AVM supplied by the branches of posterior communicating artery and posterior cerebral artery, with a nidus measuring 1.3 cm and single deep venous drainage through the internal cerebral vein. Intraoperative road-map image of the vertebral artery in lateral **(D)** and AP **(E)** views exhibit the process of navigating to the posterior cerebral artery and the posterior communicating artery, respectively. Intraoperative nonsubtracted single shot imaging from the AP **(F)** view revealing the cast of Onyx (EV3, Irvine, California). After the intervention, control DSA within the right ICA in lateral **(G)** view and vertebral artery in lateral **(H)** view highlighted anatomical exclusion of nidus. Postoperative head CT **(I)** confirmed no intracranial hemorrhage and edema. AP, anteroposterior; AVM, arteriovenous malformation; CT, computed tomography; DSA, digital subtraction angiogram; ICA, internal carotid artery; MRI, magnetic resonance imaging.

Endovascular procedures were performed using variable stiffness microcatheters under general anesthesia and systemic heparinization in a biplane angiographic unit (Figure 1). Superselective embolization of AVMs through a transfemoral route was initially performed when feeding arteries could be accessed with microcatheters, and appropriate working angles for the catheterization of the AVM’s arterial supply were recorded. The microcatheters were then used to advance the microcatheter tip as close as possible to the AVM nidus for the feeders to be filled. Once inside the nidus, permanent vessel occlusion or embolization of the AVM nidus was obtained with injection of Onyx-18 Micro Therapeutics (eV3 Inc., Irvine, California, United States) alone/ or Onyx and Glubran-2 (GEM SRL, Viareggio, Italy) into the target vessels. For patients who presented with an AVM associated with a flow-related aneurysm, treatment of the aneurysm was done first. During the injection, multiple oblique views were needed to readily recognize any inadvertent reflux from the tip of the microcatheter. As a general rule, embolization was stopped when reaching the safest volume of the AVM to be embolized. An AVM was considered cured once no early venous drainage was visible on any DSA incidence.

### Transvenous approach

**Figure 2:**
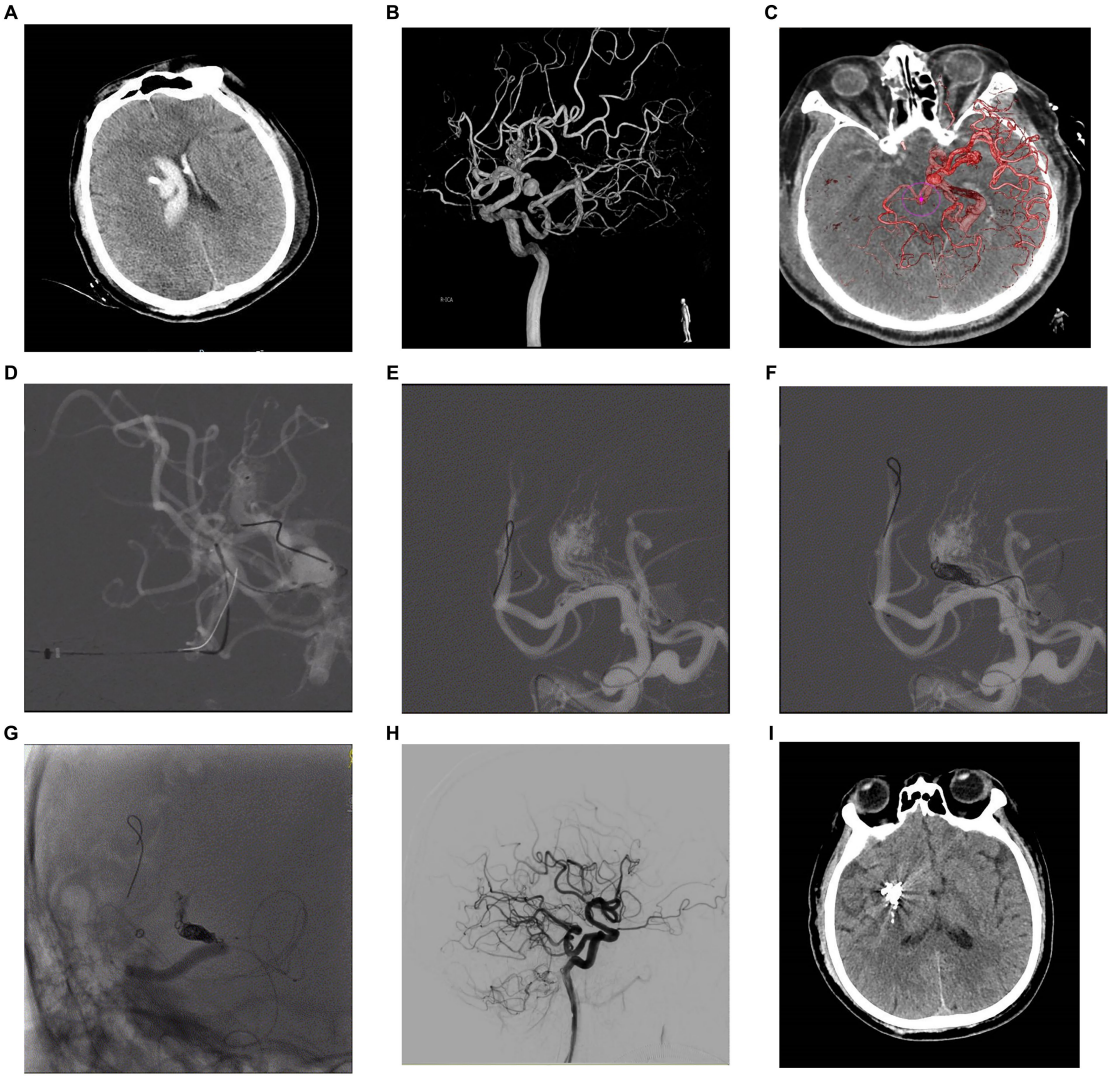
A 57-year-old male patient presented with sudden headache and acute confusion of consciousness, who was performed emergency external ventricular drainage, and, 4 weeks later, the AVM was embolized by using the TVA. Nonenhanced CT scan in axial view **(A)** showed acute hematoma in right thalamus with ventricular extravasation. Three-dimensional DSA **(B)** revealed an AVM supplied by anterior choroidal arteries and lenticulostriate arteries with a nidus measuring 1.0 cm. Venous aneurysm is presented in the venous drainage network of AVM. The deep drainage was conducted through the superior petrosus sinuses. The fusion image of three-dimensional DSA and Xper-CT **(C)** better delineated the location relationship of AVM nidus. Intraoperative road-map image in lateral view exhibits a 6F SOFIA intermediate catheter (MicroVention, Tustin, California) placed at superior petrosus sinuses and a microcatheter Apollo (EV3, Irvine, California) navigated as close as possible to the initial part of primary draining vein **(D)**, then another microcatheter Headway Duo (MicroVention, Tustin, California) was placed to near the Apollo. Its tip was positioned between the most distal marker and the detachment zone of the Apollo. A ScepterC balloon **(E)** was placed in the right middle cerebral artery in preparation for blocking the blood supply to the perforator artery. Then, coils were placed in the draining vein through the Headway Duo microcatheter to create a plug **(F)**. Intraoperative nonsubtracted single shot in AP **(G)** view showing the cast of Onyx (EV3, Irvine, California). After treatment, control DSA of the right ICA in lateral **(H)** view confirmed anatomic resolution of the lesion. Postoperative head CT **(I)** confirmed no intracranial hemorrhage and edema. AP, anteroposterior; AVM, arteriovenous malformation; CT, computed tomography; DSA, digital subtraction angiogram; ICA, internal carotid artery; TVA, transvenous approach.

Transvenous cerebral AVM embolization in the deep locations, with unfavorable arterial angioarchitecture is gaining more terrain recently due to the easier access to the nidus through the draining veins (Figure 2). However, the transvenous retrograde embolization of an AVM nidus requires feeder control from the arterial side. Under the guidance of the road map, the intermediate catheter was placed at a deep drainage vein, and a detachable tip microcatheter was navigated as close as possible to the initial part of primary draining vein, then another embolized microcatheter was placed near the detachable microcatheter. Its tip was positioned between the most distal marker and the detachment zone of the detachable microcatheter. A balloon was placed in the main artery in preparation for blocking the blood supply to the perforator artery. Then, coils were placed in the draining vein through the embolized microcatheter to create a plug to prevent the Onyx-18 Micro Therapeutics ([eV3 Inc., Irvine, California, United States) from refluxing and push more Onyx continuously into the nidus by detachable microcatheter in a manner of transvenous retrograde permeation. Therefore, in this way, it may effectively avoid filling arterial feeders and their branches to normal brain tissue. After treatment, control DSA was performed to confirm the anatomic resolution of the lesion.

### Statistical analysis

All statistical analyses were performed with the standard software (SPSS v23; SPSS, Chicago, IL). Continuous data were expressed as mean ± standard deviation (SD). The frequencies and percentages of categorical variables are reported.

## Results

### Patient population

The present study consisted of 8 female and 4 male patients, 4 to 57 years of age (mean 23.83 ± 16.51). Moreover, half of the patients were under the age of 18. The AVMs were located in the thalamus in 3 (25%) patients, in the basal ganglia in 3 (25%) patients, and in both sites of the brain in 6 (50%) patients. There were 5 AVMs located on the left side, and 7 on the right. There were 4 small (0–3 cm in diameter), 7 medium (3–6 cm in diameter), and 1 large (> 6 cm in diameter) AVMs. The AVMs ranged in size from 1.3 to 6.1 cm prior to embolization, and the mean nidus diameter was 3.32 ± 1.43 cm. None of the patients had undergone prior surgery for AVM resection, the time interval between symptom onset and treatment ranged from 6 to 115 days (mean 29.17 ± 29.30). There are 3 patients treated in the acute phase within two weeks of bleeding, and 4 patients within one month, 3 patients more than one month, only one patient later after 3 months. When graded using the Spetzler-Martin classification, 4 (33.3%) were Grade III, 7 (58.3%) were Grade IV, and 1 (8.3%) was Grade V. The thalamoperforating arteries and lenticulostriate arteries were the most common sources of arterial supply and either or both were involved in supplying 12 of the AVMs. Supply from both thalamoperforating and lenticulostriate vessels was present in 6 patients. Six AVMs were fed by the anterior choroidal artery, 7 by the posterior choroidal arteries, and 2 by the perforating branches of the posterior cerebral artery. The venous drainage is done by both the superficial and the deep venous systems. All AVMs had deep venous drainage. All but 3 cases involved a single deep vein drainage, with 2 cases having multiple deep and superficial vein drainage and 1 case having multiple deep vein drainage. The deep venous drainage was through the basal vein of Rosenthal or the internal cerebral vein to the vein of Galen, or through the superior petrosal sinus to the transverse and sigmoid sinus. Superficial veins are drained through superficial middle cerebral veins or superiorly to the superior sagittal sinus. Venous aneurysm was presented in 4 patients. All of the patients presented with bleeding at admission: four of these patients presented with an ICH, 8 ICH in combination with IVH, and no patient with SAH. Patient baseline data of AVMs are listed in Table 1 and AVMs characteristics and Clinical Outcome are described in Table 2.

**Table 1:** Patient baseline data of AVMs.

Characteristic	Value
Sex, female	8 (66.7)
Age, years, mean ± SD	23.83 ± 16.51
AVMs size, cm, mean ± SD	3.32 ± 1.43
Small (≤3 cm)	4 (33.3)
Medium (3-5 cm)	7 (58.3)
Large (>6 cm)	1 (8.3)
*Types of hemorrhage*	
ICH	4 (33.3)
ICH with IVH	8 (66.7)
Spetzler-Martin grade	
III	4 (33.3)
IV	7 (58.3)
V	1 (8.3)
Symptom onset to treatment, days	29.17 ± 29.30
*Preoperative mRS score*	
1	6 (50.0)
2	0
3	1 (8.3)
4	4 (33.3)
5	1 (8.3)
6	0
*Follow-up mRS score*	
1	5 (41.7)
2	2 (16.7)
3	2 (16.7)
4	2 (16.7)
5	0
6	1 (8.3)

Except as noted for age, AVMs size and time from symptom onset to treatment, values are reported as number(%) based on 12 patients and 12 AVMs. AVMs: arteriovenous malformations; mRS: Modified Rankin Scale; ICH: Intracerebral hemorrhage; IVH: Intraventricular hemorrhage; SD: Standard deviation.

**Table 2:** AVMs characteristics and clinical outcome.

Patient	Age (years), sex	Presentation	Initial mRS	Location	Size (cm)	Feeding artery	Spetzler martin grade	Venous drainage, No. Veins	Approach	Complications	Final mRS	Angiographic results	Staged
1	31, F	ICH	1	Left basal ganglia and thalamus	3.9	4	4	Deep, single	TAA	No	1	50%	2
2	4, F	ICH	5	Right basal ganglia and thalamus	1.3	3	3	Deep,single	TAA	No	1	Cure	2
3	17, M	ICH + IVH	4	Left basal ganglia and thalamus	3.8	3	4	Deep,single	TAA	no			
	3	Cure	2										
4	14, M	ICH + IVH	4	Left basal ganglia and thalamus	4.9	5	4	Deep,single	TAA	No	1	60%	3
5	46, F	ICH	1	Left basal ganglia	6.1	5	5	Deep and superficial, multiple	TAA	Infarction	2	40%	0
6	10, F	ICH + IVH	1	Right thalamus	2	3	3	Deep,single	TAA	No	1	Cure	0
7	57, M	ICH + IVH	1	Right basal ganglia	1	2	3	Deep,single	TVA	No	2	Cure	0
8	12, F	ICH + IVH	1	Right basal ganglia and thalamus	3.9	3	4	Deep and superficial, multiple	TAA	No	1	60%	2
9	34, F	ICH + IVH	4	Right basal ganglia and thalamus	2.9	2	3	Deep,single	TVA	No	4	Cure	0
10	32, F	ICH + IVH	3	Right thalamus	3.5	3	4	Deep,single	TVA	no	4	Cure	0
11	6, F	ICH + IVH	1	Right thalamus	3.4	4	4	Deep,multiple	TVA	Dead	6	Cure	0
12	23, M	ICH	4	Left basal ganglia	3.1	3	4	Deep,single	TAA + TVA	No	3	Cure	2

ICH, intracerebral hemorrhage; IVH, intraventricular hemorrhage; mRS, modified Rankin Scale; AVM, arteriovenous malformation; TAA, transarterial approach; TVA, transvenous approach.

### Treatment

In the acute phase of intracranial bleeding, one patient suffered a large ICH in the left basal ganglia with compression of the lateral ventricle and an approximately 13 mm midline shift. He presented with disturbance of consciousness preoperatively, and then the hematoma was evacuated on an emergency basis, without aiming for the AVM nidus. After the operation, this disturbance improved. Four patients with ICH and IVH performed external ventricular drainage. After multidisciplinary discussion, all patients were treated with endovascular embolization procedures, 7 patients were treated by the transarterial approach, 4 patients transvenous approach, and 1 patient underwent the combined approach. A single embolization procedure was performed in 6 patients (50%) and the other 6 cases (50%) were treated in a staged manner with up to three procedures.

### Complications and outcomes

One patient died of hemorrhage and diffuse brain swelling, and 4 patients presented with cerebral edema around the lesion after embolization via transvenous approach. One patient with basal ganglia AVM embolized via a transarterial approach developed postoperative cerebral infarction due to glue reflux. The AVMs were completely cured in 7 patients (58.3%) and partially obliterated in 4 patients (33.3%). The 4 patients with residual AVMs have now been fully treated by radiosurgery and are currently within the latency period. The MRS score improved at the time of last follow-up after treatment compared to their functional status at discharge. Overall, Good or excellent outcomes were obtained in 7 patients (58.3%), and poor functional outcome was observed in 5 patients (41.7%) at the last follow-up. Among the 6 patients with pre-operative neurological deficits, 1 patient had no change in their condition, 2 patients had improvement, 2 patients experienced postoperative neurological recovery, and 1 patient had deterioration. However, among the 6 patients whose neurological examination was normal pre-operatively, 3 patients remained unchanged, 3 patients had deterioration of their neurological function status, and one of them died. Of note, there was no postoperative bleeding or recurrence occurred during the follow-up.

## Discussion

Brain AVMs are vascular abnormalities that lack a capillary bed and the blood from the supplying arteries is shunted through the nidus into the draining veins. The abnormal structure of the nidus leads to high shunt flows and drainage venous hypertension. These complex lesions located in the basal ganglia and thalamus pose great challenges and difficulties for treatment. In the first place, the clinical onset of basal ganglia and thalamic AVMs occurs mostly in children and young adults and is usually hemorrhagic. In the present series, children (<18 years) constituted of 50% patients with basal ganglia and thalamic AVMs. There is no exact explanation of this epidemiological characteristic (10). Secondly, given the functional significance of their critical location, this may predispose patients to severe neurological deficits, mainly including sensory and motor impairment. The etiopathogenesis of basal ganglia and thalamus AVMs is not well understood (11), and their natural history remains unclear (12). At present, various treatment options are available for brain AVMs, including observation, microsurgery, stereotaxic radiosurgery (SRS), endovascular therapy, or a combination of these options. However, the optimal choice of treatment for these basal ganglia and thalamic lesions has not been established. Treatment selection must be tailored on the patient’s symptoms, lesion site, size, its type, arterial feeders, venous drainage, and the Spetzler-Martin grade as well as the neurologic status of the patient. In addition, the morbidity and mortality of each treatment, and long-term outcomes must be taken into account. Given the higher risk of bleeding, poor clinical outcomes and more aggressive clinical course, early treatment of these vascular lesions is rational and paramount to prevent ICH. Here in, we reviewed our experience with AVMs in the thalamus and basal ganglia to summarize the optimal treatment options and indications.

Microsurgery is considered the first-line therapy for cortically-based or non-eloquent AVMs given the immediate elimination of hemorrhage risk (13, 14). However, the surgical management of basal ganglia and thalamus AVMs is particularly challenging, because the nidus involves vital neural structures and limited operative exposure. For these lesions, the crucial to the success of such an intervention is an appropriate corridor of access (7). These AVMs are usually supplied by the deep and multiple origin of arteries, such as the lenticulostriate artery or the anterior choroidal artery, thalamoperforating arteries (from P1 posterior cerebral and posterior communicating arteries), as well as posterior choroidal arteries, which may block accessibility to supplying arteries and affect early vascular control. As a result, when resecting an AVM in the basal ganglia and thalamus, it may lead to an uncontrollable hemorrhage and damage surrounding tissues, and then cause severe disability. The choice of surgical approach was based on AVM location, arterial supply, and presence of surrounding hematoma. A hematoma often separates the nidus from surrounding tissue, enhancing the exposure of the contour and depth of the lesion and requiring less manipulation of the surrounding parenchyma. For patients with hemorrhagic brain AVMs, microsurgery can remove the hematoma and relieve the mass effect, extirpate the lesions completely, and eliminate the risk of bleeding. Consequently, the overall complete resection rate is up to 71%, with an overall surgical mortality rate of 29% (15). Of note, patients across these surgical series were carefully selected and mainly include young age, small size, and compact nidus, lesions with hematoma in the thalamus or caudate nucleus, and most importantly, a history of hemorrhage that already caused preoperative neurological deficits (7, 8, 16, 17).

However, the size of the nidus, location, arterial supply, and venous drainage are probably the most important factors for determining the feasibility and surgical risk associated with basal ganglia and thalamic AVMs (14, 18). Therefore, in order to gain a better clinical outcome, selecting suitable patients for microsurgical resection is critical. In this article, we present a series of basal ganglia and thalamic AVMs with hemorrhagic presentation, multiple origin of arterial supply, deep venous drainage, and high Spetzler-Martin grade were less likely to be treated with microsurgery. As expected, the microsurgical resection of these lesions appears to carry an increased risk of surgical morbidity and mortality.

SRS has been proposed as a good option for treating deep-seated and inaccessible small AVMs (8, 19, 20), particularly in patients with no history of hemorrhage (8). It also can be used as an assistance measure for the residual brain AVMs after the operation or endovascular treatment. The obliteration rates of AVMs in the basal ganglia and thalamus varied from 43 to 86% at 4 or 5 years, and the rates of permanent morbidity varied from 4 to 17% (6, 13). However, the primary limitation of SRS is the substantial delayed effect of nidal obliteration, and it takes 2 to 5 years to achieve satisfactory results from radiosurgery^(^^21^^)^. During the latency period between radiosurgery and obliteration, the patient is still exposed to the risks of AVM hemorrhage, which carries significant additional risk of morbidity and mortality (6, 7, 9). The basal ganglia and thalamic AVMs have shown greater resistance to radiosurgical cure compared with most other AVMs (7, 22), and permanent radiation-induced side effects are higher for these lesions (7, 22, 23). The incidence of symptomatic radiation edema is as high as 13% in basal ganglia and thalamic AVMs (8). Treatment may fail due to recurrent hemorrhage and radiation damage. Additionally, the basal ganglia and thalamus are exquisitely sensible to radiation effects, lower marginal doses are needed to avoid radiation-related complications in treatment (24). Ruptured AVMs of the basal ganglia and thalamus require more immediate protection against the risk of rehemorrhage, which makes the radiosurgical latency period problematic. In the current cases, all basal ganglia and thalamic AVMs presented with ICH, which may not be suitable for SRS directly at first, due to their hemorrhagic manifestations and relatively large nidus involved in the internal capsule and/or the thalamus and several different feeding arteries including the choroidal artery (25). In addition, the obliteration rates for medium and large AVMs would therefore be smaller, and radiosurgery would be ineffective as the primary treatment for these lesions (26).

In the early stage, transvenous approach embolization for intracranial AVMs is not prevalent, so we adopted targeted transarterial approach embolization for these lesions to reduce the risk factors of rebleeding and the nidus to a size suitable for radiosurgery. We chose SRS for the residual malformations or for the malformations that had no suitable arterial access. In our case, the 4 patients with residual AVMs have been treated by SRS and are currently within the latency period.

Endovascular embolization therapy is playing an increasingly important role in the treatment of deep-seated AVMs, due to the development of embolic materials, microguide wires, endovascular techniques, treatment strategies, and so on. It not only serves as an adjunctive therapy with microsurgery and radiosurgery or as part of multimodal brain AVMs treatment, but also as stand-alone therapy for deep AVMs. However, only a small percentage of basal ganglia and thalamic AVMs were treated by embolization alone as a curative procedure, due to the difficulty of catheterizing the small feeding perforating arteries and a high risk of complications. The general complete obliteration rate ranges between 0 and 24% and an incidence of permanent neurological deficits ranging from 10.5 to 60% (8, 14, 23, 27). For these basal ganglia and thalamus AVMs, we need a thorough evaluation of risk to get satisfactory curative rates and acceptable morbidity. In our report, we used interventional embolization to treat basal ganglia and thalamus AVMs, with a complete cure rate of 58.3% and an incidence of neurological dysfunction of 25%. Our results indicate a significant increase in the rate of anatomical elimination for such a special subset of malformations.

The endovascular embolization treatment for cerebral AVMs can be performed through transarterial anterograde or transvenous retrograde access. Transarterial basal ganglia and thalamic AVMs embolization mainly involves highly selective access of the feeding vessels and the introduction of an embolic agent that will occlude the nidus. In this way, the blood flow pressure in the malformations and the risk of bleeding can be significantly reduced. However, this method requires appropriate arterial approaches. The lesions are usually supplied by the lenticulostriates, thalamoperforators, and/ or the choroidal arteries. These vessels generally have a small intralumial diameter and arise from the parent vessel at right or acute angles, which makes superselective catheterization difficult and dangerous, and even leads to bleeding, due to the dilated perforator vessels being prone to rupture during the procedures. Hemorrhagic complications can also be due to intraoperative rupture of the brain AVM, premature venous embolization results in elevated nidus pressures, or postembolization venous stagnation in and/or around the nidus (28, 29). The causes of ischemic complication may be due to antegrade occlusion of a normal artery distal to the nidus or due to the lack of sufficient safe distance leading to the glue refluxed to retrograde occlude normal arteries (29). During operation, reflux of the embolic agent is not allowed, and a small amount of reflux will cause serious complications. For some cases, the transarterial “pressure cooker” technique was used to reduce the blood flow through the AVM, to lessen the risk of reflux inside the normal vessels, and improve the embolization rate of AVM. In our group of cases, 5 patients were treated with liquid embolism agent Onyx-18 alone, while 2 patients were treated with Onyx and Glubran-2 together via transarterial approach, of which 6 (85.7%) obtained good clinical outcome and 3 (42.9%) obtained anatomic obliteration. Only one patient developed postoperative cerebral infarction due to glue reflux. Fortunately, The patient experienced a favorable recovery. There are also those AVMs not amenable to embolization via transarterial approach, usually because of the difficulties in establishing arterial access through the small perforating feeders and the high risk of complications. Under the circumstances, a transvenous embolization approach may be the alternative option for treating these basal ganglia and thalamic AVMs. In this method, it can more easily access the nidus through the draining veins (21) effectively avoiding delivery of embolic agents via arterial feeders and preventing occlusion of arterial branches to adjacent normal brain tissue. The relative indications for transvenous embolization include small nidus sizes, single venous drainage, absence of a navigable arterial feeder, and nil transarterial access to the nidal remnant (30, 31). Latterly, 4 patients were treated by transvenous approach, because of the inaccessibility of catheterizing the arterial feeders, and the lesions were not suitable for microsurgery or radiosurgery. All of the AVMs obtained anatomic obliteration. During the procedure, the arterial microcatheter is always placed in the main feeder arteries to achieve a better view of the nidus and optimize the position of venous microcatheters by superselective angiogram. Venous microcatheter was placed as close as possible to the nidus. It is essential to maintain venous egress until the nidus was eliminated. We controlled systemic hypotension temporarily and reduced systolic blood pressure to 60-80mmhg or used a balloon to block the main arterial feeder, in order to decrease the feeding arteries’ flows and nidal pressures sufficiently. In this way, the draining vein pressure is significantly decreased, which can allow the embolic agent to retrogradely penetrate into the nidus through the draining vein. The coils were placed in the draining vein through the microcatheter next to the nidus to increase the venous resistance and prevent the Onyx from refluxing. Then, Onyx would be injected with the “plug and push” technique, until the embolic agent has penetrated through the nidus. Once full retrograde filling of the nidus with onyx and anatomic obliteration of the AVM, we detached the tip of the detachable microcatheter and retrieved it carefully. The application of the detachable microcatheters can reduce the risk of tearing the draining vein and the associated hemorrhage during the removal of the microcatheters after embolization. In this series, peri-nidal edema occurred in all patients, which may be related to impaired cerebral vasoreactivity of the perinidal tissues. We treat it by dehydration, methylprednisolone, and controlling systolic blood pressure. Only one patient with multiple deep drainage veins died of hemorrhage and diffuse brain swelling after embolization, which may be related to the elevated drainage pressures. The remaining three patients with a single deep drainage vein obtained better treatment results. Lesions with a single draining vein are better suited for the transvenous approach, while lesions with multiple venous drainage increase the risk of hemorrhagic complications (32). One patient with residual AVM after transarterial approach embolization, subsequently, we performed a second stage of transvenous interventional embolization and achieved curative elimination of the residual AVM. Of note, there was no postoperative bleeding or recurrence occurred during the follow-up. Our series of 5 patients demonstrated the feasibility, effectiveness, and high obliteration rates of transvenous embolization for basal ganglia and thalamic AVMs. Transvenous embolization may be used as a salvage treatment for cerebral AVM remnants after incomplete treatment using established current methods. We think that appropriate patient selection, accurate assessment of the malformations characteristics, and extensive experience are the key points to achieve a good clinical outcome.

### Limitations

Our study introduced a retrospective approach with inherent limitations that may cause some bias, the number of patients was small and the duration of follow-up was relatively short, so multivariate analysis could not be performed. As stated above, further studies and larger case series are necessary to assess the efficacy and durability of this treatment.

## Conclusion

The management of basal ganglia and thalamic AVMs is complicated, and treating these lesions entails considerable risks, which need multimodal and individualized treatment to improve the chances of radiographic cure and good outcomes. Endovascular therapy is a safe and effective treatment for hemorrhagic cerebral AVMs located in the basal ganglia and thalamus that are not suitable for microsurgery or radiotherapy. Our results demonstrate a high percentage of anatomic obliteration with an acceptable rate of complications in the endovascular treatment of these vasculopathies via a transarterial approach or a transvenous approach. Under the appropriate treatment strategies and choices, embolization from the venous approach is an alternative method for patients with basal ganglia and thalamus AVMs which cannot be occluded from the arterial approach.

## Data availability statement

The raw data supporting the conclusions of this article will be made available by the authors, without undue reservation.

## Ethics statement

Ethical review and approval was not required for the study on human participants in accordance with the local legislation and institutional requirements. Written informed consent from the patients/participants or patients/participants’ legal guardian/next of kin was not required to participate in this study in accordance with the national legislation and the institutional requirements. Written informed consent was obtained from the individuals or minors’ legal guardian/next of kin for the publication of any potentially identifiable images or data included in this article.

## Author contributions

All authors listed have made a substantial, direct, and intellectual contribution to the work and approved it for publication.

## Abbreviations

AVM: arteriovenous malformation
AVMs: arteriovenous malformations
DSA: digital subtraction angiography
ICH: intracerebral hemorrhage
IVH: intraventricular hemorrhage
MRI: magnetic resonance imaging
mRS: modified Rankin Scale
SAH: subarachnoid hemorrhage
SRS: stereotaxic radiosurgery

## References

[1] Osbun JW Reynolds MR Barrow DL. Arteriovenous malformations: epidemiology, clinical presentation, and diagnostic evaluation. Handb Clin Neurol. (2017) 143:25–9. doi: 10.1016/B978-0-444-63640-9.00003-5 28552148

[2] Dinc N Won SY Brawanski N Quick-Weller J Herrmann E Seifert V. Predictive variables for the presence of vascular malformations as the cause of basal ganglia hemorrhages. Neurosurg Rev. (2020) 43:223–9. doi: 10.1007/s10143-018-1040-3, PMID: 30334172

[3] Fleetwood IG Marcellus ML Levy RP Marks MP Steinberg GK. Deep arteriovenous malformations of the basal ganglia and thalamus: natural history. J Neurosurg. (2003) 98:747–50. doi: 10.3171/jns.2003.98.4.0747, PMID: 12691399

[4] Madhugiri VS Teo MKC Westbroek EM Chang SD Marks MP Do HM. Multimodal management of arteriovenous malformations of the basal ganglia and thalamus: factors affecting obliteration and outcome. J Neurosurg. (2018) 131:410–9. doi: 10.3171/2018.2.JNS172511, PMID: 30117771

[5] Potts MB Young WL Lawton MT Project UBAS. Deep arteriovenous malformations in the basal ganglia, thalamus, and insula: microsurgical management, techniques, and results. Neurosurgery. (2013) 73:417–29. doi: 10.1227/NEU.0000000000000004 23728451 PMC4123817

[6] Nagy G Major O Rowe JG Radatz MW Hodgson TJ Coley SC. Stereotactic radiosurgery for arteriovenous malformations located in deep critical regions. Neurosurgery. (2012) 70:1458–71. doi: 10.1227/NEU.0b013e318246a4d0, PMID: 22186841

[7] Gross BA Duckworth EA Getch CC Bendok BR Batjer HH. Challenging traditional beliefs: microsurgery for arteriovenous malformations of the basal ganglia and thalamus. Neurosurgery. (2008) 63:393–11. doi: 10.1227/01.NEU.0000316424.47673.03 18812951

[8] Sasaki T Kurita H Saito I Kawamoto S Nemoto S Terahara A. Arteriovenous malformations in the basal ganglia and thalamus: management and results in 101 cases. J Neurosurg. (1998) 88:285–92. doi: 10.3171/jns.1998.88.2.0285, PMID: 9452237

[9] Kiran NA Kale SS Kasliwal MK Vaishya S Gupta A Singh Sharma M. Gamma knife radiosurgery for arteriovenous malformations of basal ganglia, thalamus and brainstem—a retrospective study comparing the results with that for AVMs at other intracranial locations. Acta Neurochir. (2009) 151:1575–82. doi: 10.1007/s00701-009-0335-0, PMID: 19415175

[10] Nicolato A Foroni R Crocco A Zampieri PG Alessandrini F Bricolo A. Gamma knife radiosurgery in the management of arteriovenous malformations of the basal ganglia region of the brain. Minimal Invas Neurosurg. (2002) 45:211–23. doi: 10.1055/s-2002-36200, PMID: 12494356

[11] Chen W Choi EJ McDougall CM Su H. Brain arteriovenous malformation modeling, pathogenesis, and novel therapeutic targets. Transl Stroke Res. (2014) 5:316–29. doi: 10.1007/s12975-014-0343-0, PMID: 24723256 PMC4081044

[12] Pollock BE. The alchemy of brain arteriovenous malformation management. World Neurosurg. (2015) 83:337–8. doi: 10.1016/j.wneu.2014.05.008, PMID: 24813130

[13] Chen CJ Kearns KN Ding D Kano H Mathieu D Kondziolka D. Stereotactic radiosurgery for arteriovenous malformations of the basal ganglia and thalamus: an international multicenter study. J Neurosurg. (2019) 132:122–31. doi: 10.3171/2018.8.JNS182106, PMID: 30641831

[14] Cheng CH Crowley RW Yen CP Schlesinger D Shaffrey ME Sheehan JP. Gamma knife surgery for basal ganglia and thalamic arteriovenous malformations. J Neurosurg. (2012) 116:899–08. doi: 10.3171/2011.12.JNS11542 22264181

[15] Potts MB Jahangiri A Jen M Sneed PK McDermott MW Gupta N. Deep arteriovenous malformations in the basal ganglia, thalamus, and insula: multimodality management, patient selection, and results. World Neurosurg. (2014) 82:386–94. doi: 10.1016/j.wneu.2014.03.033, PMID: 24657255 PMC4169357

[16] Patel J Feghali J Yang W Rapaport S Gami A Sattari SA. Comparison of management approaches in deep-seated intracranial arteriovenous malformations: does treatment improve outcome? J Clin Neurosci Off J Neurosurg Soc Aust. (2021) 92:191–6. doi: 10.1016/j.jocn.2021.08.010, PMID: 34509251

[17] Sasaki T Kurita H Kawamoto S Nemoto S Kirino T Saito I. Clinical outcome of radiosurgery, embolization and microsurgery for AVMs in the thalamus and basal ganglia. J Clin Neurosci Off J Neurosurg Soc Aust. (1998) 5:95–7. doi: 10.1016/S0967-5868(98)90023-6, PMID: 18639112

[18] Pasqualin A Barone G Cioffi F Rosta L Scienza R Da Pian R. The relevance of anatomic and hemodynamic factors to a classification of cerebral arteriovenous malformations. Neurosurgery. (1991) 28:370–9. doi: 10.1227/00006123-199103000-00006, PMID: 2011218

[19] Link TW Winston G Schwarz JT Lin N Patsalides A Gobin P. Treatment of Unruptured brain arteriovenous malformations: a single-center experience of 86 patients and a critique of the a randomized trial of Unruptured brain arteriovenous malformations (ARUBA) trial. World Neurosurg. (2018) 120:e1156–62. doi: 10.1016/j.wneu.2018.09.025, PMID: 30218805

[20] Daou BJ Palmateer G Thompson BG Maher CO Hayman JA Lam KL. Stereotactic radiosurgery for brain arteriovenous malformations: evaluation of obliteration and review of associated predictors. J Stroke Cerebrovasc Dis. (2020) 29:104863. doi: 10.1016/j.jstrokecerebrovasdis.2020.104863, PMID: 32689634

[21] Mendes GA Silveira EP Caire F Boncoeur Martel MP Saleme S Iosif C. Endovascular Management of Deep Arteriovenous Malformations: single institution experience in 22 consecutive patients. Neurosurgery. (2016) 78:34–41. doi: 10.1227/NEU.0000000000000982 26317676

[22] Andrade-Souza YM Zadeh G Scora D Tsao MN Schwartz ML. Radiosurgery for basal ganglia, internal capsule, and thalamus arteriovenous malformation: clinical outcome. Neurosurgery. (2005) 56:56–64. doi: 10.1227/01.NEU.0000145797.35968.ED, PMID: 15617586

[23] Pollock BE Gorman DA Brown PD. Radiosurgery for arteriovenous malformations of the basal ganglia, thalamus, and brainstem. J Neurosurg. (2004) 100:210–4. doi: 10.3171/jns.2004.100.2.0210 15086226

[24] Kano H Kondziolka D Flickinger JC Yang HC Flannery TJ Niranjan A. Stereotactic radiosurgery for arteriovenous malformations, part 4: management of basal ganglia and thalamus arteriovenous malformations. J Neurosurg. (2012) 116:33–43. doi: 10.3171/2011.9.JNS11175, PMID: 22077451

[25] Matsushima T Fukui M Kitamura K Fujii K Hasuo K. Arteriovenous malformations in the basal ganglia. Neurol Med Chir. (1988) 28:49–56. doi: 10.2176/nmc.28.49 2455243

[26] Lawton MT Hamilton MG Spetzler RF. Multimodality treatment of deep arteriovenous malformations: thalamus, basal ganglia, and brain stem. Neurosurgery. (1995) 37:29–35. doi: 10.1097/00006123-199507000-00004, PMID: 8587687

[27] Pollock BE Flickinger JC. A proposed radiosurgery-based grading system for arteriovenous malformations. J Neurosurg. (2002) 96:79–85. doi: 10.3171/jns.2002.96.1.0079, PMID: 11794608

[28] Stemer AB Bank WO Armonda RA Liu AH Herzig DW Bell RS. Acute embolization of ruptured brain arteriovenous malformations. J Neurointervent Surg. (2013) 5:196–00. doi: 10.1136/neurintsurg-2011-010214 22406978

[29] Li G Wang G Yu J Hou K Yu J. Regression of a symptomatic varix after transarterial embolization of a brain arteriovenous malformation: a case report and literature review. Medicine. (2019) 98:e18418. doi: 10.1097/MD.0000000000018418, PMID: 31876715 PMC6946530

[30] Zhang W Wei H Tian Q Han S Han W Guo Y. Efficacy and safety of embolization for arteriovenous malformations of the basal ganglia and thalamus via the transarterial approach. Ann Trans Med. (2022) 10:306. doi: 10.21037/atm-22-384, PMID: 35433967 PMC9011271

[31] Massoud TF Hademenos GJ. Transvenous retrograde nidus sclerotherapy under controlled hypotension (TRENSH): a newly proposed treatment for brain arteriovenous malformations-concepts and rationale. Neurosurgery. (1999) 45:351–66. doi: 10.1097/00006123-199908000-00031, PMID: 10449081

[32] De Sousa JMB Iosif C Sganzerla LZ Rafie AN Borodetsky V Rouchaud A. Selection of patients for treatment of brain arteriovenous malformations by the Transvenous approach: relationship with venous anatomy and risk of hemorrhagic complications. AJNR Am J Neuroradiol. (2020) 41:2311–6. doi: 10.3174/ajnr.A6810, PMID: 33122201 PMC7963238

